# Growth Differentiation Factor-15 in Acute Coronary Syndromes: Prognostic Value and Barriers to Clinical Implementation

**DOI:** 10.3390/ijms27167093

**Published:** 2026-08-07

**Authors:** Michal Pruc, Maciej Maslyk, Andrzej Bielski, Milosz J. Jaguszewski, Lukasz Szarpak

**Affiliations:** 1 Institute of Medical Sciences, The John Paul II Catholic University of Lublin, 20-708 Lublin, Poland; 2 Institute of Biological Sciences, The John Paul II Catholic University of Lublin, 20-708 Lublin, Poland; 3 Clinical Department of Rheumatology, Provincial Integrated Hospital in Plock, 09-400 Plock, Poland; 4 Collegium Medicum, The Mazovian Academy in Plock, 09-402 Plock, Poland; 5 First Department of Cardiology, Medical University of Gdansk, 80-214 Gdansk, Poland; 6 Henry JN Taub Department of Emergency Medicine, Baylor College of Medicine, Houston, TX 77030, USA

**Keywords:** acute coronary syndrome, growth differentiation factor-15, high-sensitivity troponin, GRACE score, bleeding, dual antiplatelet therapy, risk stratification

## Abstract

High-sensitivity cardiac troponin has made the diagnosis of myocardial infarction (MI) faster and more precise, but it does not measure the broader biological vulnerability that often determines the outcome after an acute coronary syndrome (ACS). Growth differentiation factor-15 (GDF-15) is induced by ischemic stress, inflammation, oxidative injury, renal dysfunction, metabolic disease, and ageing. This biology explains its appeal in ACS, but also its diagnostic limitation: GDF-15 is not cardiac-specific and should not be used as an alternative to electrocardiography and high-sensitivity troponin algorithms for early MI diagnosis. Its better supported role is prognostic. Across emergency department chest pain cohorts, non-ST elevation MI, ST elevation MI, post-ACS trial populations, and serial biomarker studies, higher GDF-15 concentrations are most consistently associated with all-cause mortality, cardiovascular mortality, heart failure, and major bleeding, while associations with recurrent ischemic events alone are less specific. The key unresolved issue is incremental clinical value. GDF-15 may improve discrimination and reclassification beyond clinical predictors, troponin, natriuretic peptides, renal function and GRACE or GRACE 2.0 in selected settings, but statistical association is not equivalent to clinical utility. Its possible role in bleeding risk estimation and antithrombotic benefit–risk assessment is clinically important, especially after the PLATO biomarker analyses, yet routine GDF-15-guided dual antiplatelet therapy decisions remain unsupported. Future implementation requires validated thresholds, calibration, decision curve evidence, health economic evaluation, and trials in which GDF-15-guided management changes care and improves outcomes.

## 1. Introduction

The contemporary acute coronary syndrome (ACS) pathway is built around rapid clinical assessment, electrocardiography (ECG), high-sensitivity cardiac troponin (hs-cTn), and structured risk assessment [1]. European Society of Cardiology (ESC) and American College of Cardiology (ACC)/American Heart Association (AHA) recommendations reinforce this diagnostic architecture because hs-cTn is the best-validated biomarker of myocardial injury and because early treatment decisions must be time-critical, reproducible, and safe [2,3,4]. Yet a troponin-based diagnosis does not fully describe the patient in front of the clinician. It does not quantify frailty, inflammatory burden, renal reserve, subclinical heart failure (HF), vascular aging, bleeding susceptibility, or post-discharge resilience [5,6]. Those domains often explain why two patients with a similar troponin curve and angiographic result have very different outcomes [5,7]. Growth differentiation factor-15 (GDF-15) occupies this residual risk space [5,6,8]. The most defensible clinical thesis is not that GDF-15 improves the biochemical diagnosis of infarction, but that it may identify patients whose risk is biologically disproportionate to their apparent index event [7]. This is a diagnostic limitation, but also a prognostic opportunity [7]. Current ACS guidelines still rely on clinical risk models such as GRACE or GRACE 2.0 to estimate mortality and guide the intensity of care [1,9]. The implementation gap is whether a stress response biomarker can be integrated into those pathways without simply adding another number [10,11]. Most available evidence establishes an association; the unresolved question is whether GDF-15 can be operationalized into decisions [6]. Compared with earlier broad reviews of GDF-15 in cardiovascular disease, the present review focuses specifically on its position within contemporary ACS pathways and separates three questions that are frequently conflated: diagnostic performance for index myocardial infarction, prognostic performance after suspected or confirmed ACS, and evidence of clinical actionability. It additionally integrates newer evidence from high-sensitivity troponin-era emergency department cohorts, GRACE 2.0 models, bleeding risk analyses, serial biomarker trajectories, and contemporary implementation frameworks [5,6,7,11].

The aim of this state-of-the-art clinical review is therefore to define the clinically defensible position of GDF-15 in ACS by separating its diagnostic limitations from its prognostic and bleeding risk utility, evaluating its incremental value beyond hs-cTn and GRACE/GRACE 2.0, and outlining the evidentiary steps required before GDF-15 can be used to guide management rather than merely describe risk. Accordingly, the central knowledge gap framing this review is not whether GDF-15 is associated with adverse outcomes after ACS, but whether measuring it changes a clinically relevant decision and, ultimately, improves patient outcomes.

## 2. Methods

This narrative review used a targeted literature selection strategy designed to support clinical interpretation rather than formal meta-analysis. PubMed/MEDLINE, Web of Science, and Scopus were searched from database inception to 25 June 2026, supplemented by guideline documents and reference tracking from major ACS biomarker studies. Search terms combined “GDF-15” or “growth differentiation factor-15” with “acute coronary syndrome”, “ACS”, “myocardial infarction”, “MI”, “Non-ST-Elevation Myocardial Infarction”, “NSTEMI”, “ST-Elevation Myocardial Infarction”, “STEMI”, “unstable angina”, “high-sensitivity troponin”, “GRACE”, “GRACE 2.0”, “bleeding”, “dual antiplatelet therapy”, “DAPT”, “emergency department”, “ED”, “serial measurements”, and “risk stratification”.

Priority was given to contemporary guideline documents, prospective observational cohorts, biomarker substudies of randomized trials, individual participant and aggregate data meta-analyses, and studies reporting clinically interpretable measures of diagnostic, prognostic, or therapeutic risk performance. Particular attention was paid to studies evaluating discrimination, calibration, reclassification, incremental value beyond established clinical risk scores, and proposed clinical thresholds. Studies addressing bleeding risk, antithrombotic treatment intensity, mortality, recurrent myocardial infarction (MI), HF, and composite cardiovascular endpoints were prioritized where they clarified the potential clinical role of GDF-15 in ACS care.

When multiple publications originated from the same cohort or addressed substantially overlapping clinical questions, preference was given to the largest, most recent, and methodologically most complete analysis. Separate reports from the same study program were retained when they evaluated distinct endpoints, sampling time points, treatment interactions, patient subgroups, or serial biomarker trajectories. Study selection was therefore purposive and question-driven: studies were retained when they directly informed diagnosis, prognosis, bleeding risk, serial assessment, threshold calibration, or implementation, rather than to achieve exhaustive inclusion of every report mentioning GDF-15.

Eligible clinical evidence comprised full-text human studies of adults with suspected, confirmed, or recently stabilized ACS that measured circulating GDF-15 and reported diagnostic performance, mortality, recurrent ischemic events, HF, bleeding, incremental model performance, serial trajectories, thresholds, or implementation-relevant outcomes. Biomarker substudies of randomized trials, prospective and retrospective cohorts, relevant meta-analyses, and guideline documents were eligible. Mechanistic studies were retained only when they directly informed GDF-15 synthesis, processing, receptor biology, assay interpretation, or multisystem biological context. Case reports, conference abstracts without adequate full-text data, studies without a circulating GDF-15 measurement, non-relevant disease populations, and duplicate or substantially overlapping reports were excluded; overlapping publications were retained only when they addressed distinct endpoints, sampling times, treatment interactions, or patient subgroups.

Candidate publications were assessed iteratively against the predefined clinical questions. Final retention reflected direct relevance, methodological completeness, and a contribution not duplicated by a more informative report. Because this was a purposive narrative review, study selection was question-driven rather than exhaustive [12]. Accordingly, the review does not claim exhaustive study capture, and no PRISMA flow diagram, dual independent screening, or formal numerical risk-of-bias score was used.

Mechanistic and molecular studies were included selectively when they helped explain the biological interpretation of circulating GDF-15, including its regulation by inflammation, oxidative stress, tissue injury, aging, renal dysfunction, and cardiometabolic disease. Small mechanistic studies were used only to frame biological plausibility and were not treated as primary evidence for clinical performance. Reviews were used to contextualize the field but were not considered primary sources for diagnostic, prognostic, or treatment stratification estimates.

For interpretation, the clinical evidence was arranged in a qualitative hierarchy rather than treated as equally persuasive. Greatest weight was assigned to individual participant meta-analyses, prospectively collected biomarker substudies of randomized trials, and large prospective multicenter cohorts with contemporary comparators and external validation. Moderate weight was assigned to smaller prospective cohorts and secondary trial analyses, whereas small single-center, retrospective, exploratory, or incompletely validated studies were used primarily for hypothesis generation.

Critical appraisal was qualitative and considered study participation and representativeness, attrition, biomarker and outcome measurement, confounding, statistical analysis, reporting, model applicability, and external validation, consistent with domains described for prognostic factor and prediction model research [13,14].

Where possible, numerical data were extracted at the study level, including cohort size, clinical setting, ACS subtype, endpoint definition, sampling window, assay platform, follow-up duration, hazard ratios or odds ratios, area under the curve, calibration measures, net reclassification indices, decision thresholds, and incremental value beyond established predictors such as high-sensitivity cardiac troponin and GRACE-based risk models. When a metric was not reported or could not be unambiguously extracted from the article record, it was marked as not reported rather than imputed.

The narrative review deliberately separates diagnostic evidence from prognostic and therapeutic risk evidence, because combining these domains can obscure the principal clinical question: GDF-15 appears less suited as a primary diagnostic marker for MI than as a marker of vulnerability, comorbidity burden, bleeding risk, and long-term adverse outcomes in patients with suspected or confirmed ACS. Formal pooling was not performed because eligible studies differed substantially in population selection, ACS phenotype, timing of blood sampling, assay platform, comparator biomarkers, endpoint definitions, follow-up duration, and intended clinical use case.

## 3. Biology of GDF-15 in Ischemia, Inflammation, Aging and Frailty

GDF-15, originally described as macrophage inhibitory cytokine-1, is a divergent member of the transforming growth factor-β superfamily [15,16,17].

GDF-15 is synthesized as a pre-proprotein containing an N-terminal signal peptide, a pro-domain, and a C-terminal mature domain. Following dimerization, furin-like proprotein convertases cleave the precursor at a conserved RXXR motif, releasing a mature, approximately 25 kDa, disulfide-linked homodimer [17,18]. Processing may be incomplete, and secreted pro-GDF-15 can associate with the extracellular matrix through its pro-domain and form local stromal stores [18]. Heterogeneity in precursor processing and in the relative abundance of pro- and mature forms may therefore contribute to differences in analyte availability and assay recognition. This molecular-form heterogeneity should be distinguished from sequence-dependent epitope effects associated with rs1058587, which represent a separate potential source of between-assay variability [19].

GDF-15 is also regulated by the integrated stress response (ISR). Stress-responsive phosphorylation of eukaryotic translation initiation factor 2α (eIF2α) suppresses global protein synthesis while favoring translation of activating transcription factor 4 (ATF4), which, together with C/EBP homologous protein (CHOP/DDIT3), can directly induce GDF-15 transcription. Experimental activation of this eIF2α-ATF4-CHOP program during mitochondrial or endoplasmic reticulum stress and nutrient stress increases GDF-15 expression and secretion [20]. This pathway provides a molecular link between cellular energetic stress and the circulating GDF-15 signal.

The GDF-15-GFRAL-RET axis distinguishes the established endocrine actions of GDF-15 from canonical TGF-β signaling. Circulating GDF-15 binds GFRAL, which recruits RET as an obligatory coreceptor and activates RET-dependent intracellular signaling, including the ERK and AKT pathways [21,22,23]. GFRAL expression is concentrated in the area postrema and nucleus tractus solitarius, where this pathway mediates anorexia, aversive feeding responses, and weight loss during systemic illness [21,22,23]. The established endocrine pathway is therefore distinct from the conventional TβRII/ALK-SMAD receptor system, and no validated cognate receptor mediating direct actions of circulating GDF-15 in cardiomyocytes or the vascular wall has been established.

**Figure 1:**
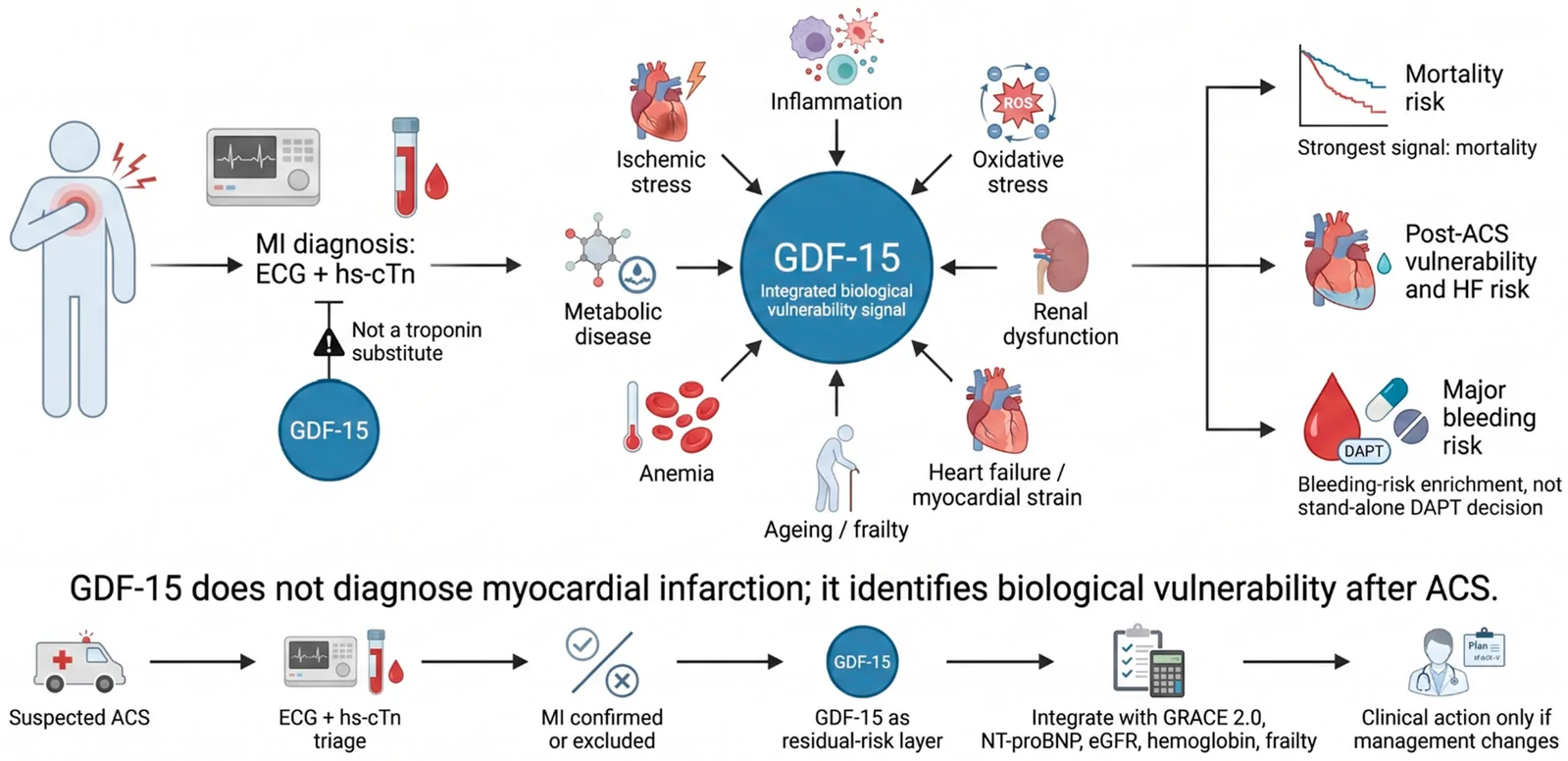
GDF-15 as a biology-of-risk biomarker in ACS. Note: Conceptual diagram showing convergence of acute ischemia–reperfusion injury, oxidative stress, inflammation, endothelial activation, renal dysfunction, heart failure, metabolic disease, aging, anemia, malignancy, and frailty on circulating GDF-15. The pathways are presented to summarize biological plausibility and reported clinical associations; they are not supported by equivalent levels of evidence and should not be interpreted as validated causal or management pathways. The diagram is informed by the mechanistic and clinical evidence reviewed in Section 3 and Section 4; it does not assign equivalent evidentiary strength or causality to each pathway.

Under basal conditions, its expression is low in most tissues, whereas circulating concentrations increase markedly in response to cellular stress [16,24]. GDF-15 is induced by hypoxia, oxidative stress, inflammation, mitochondrial dysfunction, mechanical strain, and tissue injury [6,15]. Its promoter contains p53-responsive elements, linking GDF-15 to DNA damage, cellular senescence, and biological aging; however, induction may also occur through p53-independent pathways, including NF-κB, EGR-1, and HIF-1α signaling [15,25]. This broad regulation explains why GDF-15 behaves as an integrated stress response biomarker rather than a disease-specific signal [6,26]. The multisystem biology underlying this interpretation is summarized conceptually in Figure 1. Figures are literature-informed conceptual syntheses intended to organize the reviewed evidence; they are not causal models, evidence-based recommendations, or outcome-validated management algorithms.

In the cardiovascular system, GDF-15 may be produced by cardiomyocytes exposed to oxidative stress, ischemia–reperfusion injury, angiotensin II, inflammatory cytokines, and mechanical overload [26,27,28]. Additional sources include macrophages, endothelial cells, vascular smooth muscle cells, and adipocytes, particularly under inflammatory or metabolic stress [16,26]. Experimental studies support a role for GDF-15 in myocardial stress adaptation. In the study by Kempf et al., GDF-15 was strongly induced by nitrosative stress in cultured cardiomyocytes and upregulated during simulated ischemia–reperfusion [27]. GDF-15-deficient mice exposed to transient coronary artery ligation developed larger infarcts and more cardiomyocyte apoptosis than wild-type controls, while recombinant GDF-15 protected cultured cardiomyocytes through PI3K/Akt-dependent pro-survival signaling [27]. Because GFRAL is restricted to the hindbrain in the established endocrine pathway, this PI3K/Akt-dependent cardioprotection should be interpreted as a putative local, GFRAL-independent effect whose receptor-level mechanism remains unresolved, rather than as evidence of myocardial GFRAL signalling [21,22,23,27]. These findings provide mechanistic plausibility for the association between GDF-15 and adverse cardiovascular outcomes, but they do not establish GDF-15 as a cardiac-specific marker or as a therapeutic target in ACS [27,29].

The central clinical limitation is that GDF-15 is not released from a single organ or a single pathological process. Circulating concentrations are influenced by age, renal function, diabetes, HF, anemia, smoking, systemic inflammation, malignancy and frailty [6,30,31]. Population-level data illustrate this clearly: median GDF-15 concentrations rise from approximately 537 pg/mL in men younger than 30 years to approximately 2152 pg/mL in men aged 80 years or older, while the 97.5th percentile increases from approximately 1135 pg/mL to 5972 pg/mL across the same age range [30]. In the Framingham Offspring Study, clinical correlates including age, smoking, diabetes, renal function and nonsteroidal anti-inflammatory drug (NSAID) use explained 38% of interindividual variation in GDF-15, with genetic factors accounting for a comparable proportion of residual variability [31]. Therefore, a value that is highly abnormal in a younger patient with preserved renal function may be less specific in an elderly patient with chronic kidney disease (CKD), anemia, and established HF [26,30].

Frailty and reduced physiological reserve are also closely related to the GDF-15 signal. A systematic review of 35 studies reported consistent associations between higher GDF-15 concentrations, poorer physical performance, and greater frailty severity, although associations with sarcopenia were less uniform [32]. In the FRASNET cohort, higher baseline GDF-15 was associated with subsequent frailty over six years and showed discrimination for frailty using the Fried Phenotype (AUC 0.85) and Clinical Frailty Scale (AUC 0.96), as well as for malnutrition (AUC 0.94) [33]. In the BIOFRAIL study of mobility-limited older adults, median GDF-15 was 2252 pg/mL in frail individuals compared with 1438 pg/mL in non-frail individuals, with an optimal frailty cut-off of 2047 pg/mL and an AUC of 0.681 [34]. These data support the interpretation of GDF-15 as a marker of systemic vulnerability rather than coronary injury alone [32,35].

The GFRAL-RET pathway provides interpretive context for cachexia and systemic illness across adult and pediatric disease settings [36,37]. This pathway is clinically targetable in cancer cachexia, as shown by phase 2 data with the GDF-15 inhibitor ponsegromab, but this evidence should not be extrapolated to ACS treatment [38]. Its relevance in ACS is interpretive: elevated GDF-15 may identify patients in whom the coronary event occurs on a background of aging, catabolic stress, inflammation, renal dysfunction, malnutrition, or reduced physiological reserve [6,7].

This biology explains the central paradox of GDF-15 in ACS. Because it is induced by ischemia, inflammation, oxidative stress, renal impairment, metabolic disease, mechanical overload, and biological aging, GDF-15 is well suited to capture mortality risk and global vulnerability [5,6]. At the same time, this same breadth limits its diagnostic specificity for acute MI (AMI) [7]. An individual patient meta-analysis including 53,486 patients showed that GDF-15 added prognostic information for cardiovascular death and HF hospitalization across atherosclerotic cardiovascular disease, but its association with future MI was not significant in the acute ACS setting, with a hazard ratio of 0.98 and 95%, and CI of 0.90–1.06 [5]. Similarly, in older adults from ARIC, the highest GDF-15 quartile was associated with diabetes, atherosclerotic cardiovascular disease, HF, elevated hs-cTnT, and elevated N-terminal pro-B-type natriuretic peptide (NT-proBNP), illustrating the breadth of disease captured by a single elevated value [39].

Accordingly, GDF-15 should be interpreted in ACS as a biomarker of biological risk, not as a biomarker of myocardial necrosis [6,7]. A high value in non-ST elevation MI (NSTEMI) may reflect the infarct, but it may also reflect CKD, occult or overt HF, systemic inflammation, malignancy, anemia, frailty, or vascular senescence [26,39]. This non-specificity is a limitation when the clinical question is early AMI rule-in or rule-out, where high-sensitivity cardiac troponin remains the appropriate biomarker [7]. This broad biological responsiveness may become advantageous when the clinical question concerns broader risk estimation after ACS [5,6]. If GDF-15 is measured in a research or selected clinical context, an elevated value should be interpreted as a non-specific vulnerability signal and may justify clinically driven assessment for renal dysfunction, ventricular dysfunction, anemia, frailty, or systemic disease [6,40]. It should not by itself determine diagnosis, disposition, or treatment [7].

## 4. Analytical and Pre-Analytical Considerations

Most clinical ACS studies report GDF-15 in pg/mL or ng/L, which are numerically equivalent [26,41]. Assays have included research immunoassays, electrochemiluminescence platforms, and trial-specific central-laboratory methods [37,41,42]. This matters because the literature mixes absolute cut-offs, tertiles, quartiles, and log-transformed continuous models [5,43]. Sampling has also varied: at emergency presentation, after diagnostic work-up, at randomization, at one month after ACS, or serially after discharge [44,45,46]. GDF-15 has low short-term biological variability in stable post-ACS trajectories, but that does not make a single admission value interchangeable with a convalescent measurement [44,46,47].

Future assay validation studies should explicitly evaluate whether antibody pairs distinguish the mature circulating dimer from precursor or pro-domain-containing forms and should report the epitopes targeted by the assay. Molecular-form heterogeneity and sequence-dependent epitope variation are analytically distinct and should not be treated as interchangeable explanations for between-platform disagreement [17,18]. There is no universal ACS-specific clinical cut-off. Historical research categories below 1200 pg/mL, 1200–1800 pg/mL, and above 1800 pg/mL are useful for orientation, particularly in non-S elevation acute coronary syndrome (NSTE-ACS) and trial cohorts, but they should not be treated as decision thresholds detached from age, estimated glomerular filtration rate (eGFR), HF, diabetes, inflammatory disease, cancer, or frailty [5,8,26,30,43,48,49]. Any implementation protocol must specify the platform, sampling time, clinical context, and the management action linked to the result [26].

For clinical implementation, the laboratory report would need to do more than provide a concentration. It should state the assay, unit, timing relative to symptom onset or randomization, and an interpretive caution for age, renal function, and HF [26,47]. A baseline value drawn during shock, sepsis, acute kidney injury, or decompensated HF should not be interpreted like a value obtained after clinical stabilization [44,45]. This is one reason serial studies are informative: they separate the acute inflammatory and hemodynamic perturbation of the index event from the patient’s persistent post-discharge risk phenotype [44,46].

## 5. Diagnostic Value in Suspected ACS and AMI

The diagnostic question is straightforward—does GDF-15 identify AMI better than ECG and hs-cTn algorithms? It does not. In the early international chest pain study by Schaub et al., the AUC for AMI was 0.69 for GDF-15 versus 0.96 for hs-cTnT, whereas mortality discrimination was much stronger [50]. The contemporary APACE analysis makes the same point in the hs-cTn era. In 4779 unselected emergency department patients with acute chest pain, the primary diagnostic endpoint was NSTE-MI at presentation. GDF-15 showed only modest diagnostic discrimination for index MI/NSTE-MI (AUC 0.69), but high prognostic discrimination for all-cause mortality at 90 days and five years (C-index 0.86 and 0.84, respectively) [7]. This is the clearest quantitative expression of the review’s central thesis: diagnostic limitation but prognostic opportunity. This apparent contradiction reflects the biology of GDF-15: because it is induced by multisystem stress and comorbidity rather than cardiomyocyte necrosis alone, its broad responsiveness reduces specificity for index MI while strengthening its association with longer-term mortality and systemic vulnerability [6,7,26]. The key ED and ACS cohorts supporting this distinction are listed in Table 1.

The biology is consistent with this pattern. Early AMI diagnosis requires a marker of myocardial injury with appropriate kinetics and cardiac specificity [62]. GDF-15 rises in many acute and chronic stress states and cannot reliably distinguish plaque rupture, demand ischemia, renal dysfunction, infection, HF, or malignancy [6,26]. Very early presenters, older adults, women, patients with CKD, and multimorbid patients are exactly the groups in whom a non-specific stress marker is most likely to be elevated for reasons other than acute coronary thrombosis [7,30]. GDF-15 may still be useful after troponin-based triage, particularly when a patient has normal or low hs-cTn but is not biologically at low risk [52,63].

A further practical problem is that the diagnostic literature rarely provides threshold-level evidence suitable for clinical rule-in or rule-out decisions [62,64]. As summarized in Table 2, sensitivity, specificity, positive predictive value and negative predictive value are often unavailable for a pre-specified GDF-15 threshold, or are not applicable because the study addressed prognosis rather than index MI diagnosis [50,51,65]. This distinction is clinically important: an ED diagnostic algorithm requires threshold-level safety, not only a statistically significant association or an AUC [62]. Accordingly, the potential role of GDF-15 begins after established hs-cTn-based diagnostic triage, as a marker of residual risk rather than index MI.

## 6. Prognostic Value in Confirmed ACS

The strongest evidence for GDF-15 is prognostic and endpoint-specific. In NSTE-ACS, early studies showed graded mortality risk across GDF-15 categories, with particularly high one-year mortality above 1800 pg/mL [49]. In ST-elevation MI (STEMI) and post-MI cohorts, higher GDF-15 concentrations were similarly associated with greater risks of death, HF, and composite cardiovascular events [54,55]. In PROVE IT-TIMI 22, patients stabilized after ACS with GDF-15 above 1800 pg/mL had a higher two-year risk of death or MI than those below 1200 pg/mL, and the risks of death and HF were also elevated [8].

The endpoint nuance is important. GDF-15 is most consistent for all-cause mortality, cardiovascular mortality, and global vulnerability. In PLATO, baseline GDF-15 was independently associated with cardiovascular death, all-cause death, spontaneous MI, stroke, and major non-coronary artery bypass grafting (CABG) bleeding, but the relative signal was stronger for death and bleeding than for recurrent MI [59]. Individual participant data and aggregate data meta-analyses support this hierarchy: GDF-15 is a broad cardiovascular risk marker, yet recurrent ischemic events alone are less cleanly captured in ACS once established clinical and biomarker predictors are included [5,58,66].

HF is a particularly plausible domain. GDF-15 integrates myocardial stress, renal dysfunction, aging, and systemic inflammation, all of which shape post-ACS decompensation. Chest pain cohorts in which index AMI was excluded have shown that high GDF-15 is associated with subsequent HF hospitalization as well as death, emphasizing that meeting validated hs-cTn rule-out criteria does not necessarily imply low long-term biological risk [52]. Stroke data are less mature and should be interpreted as part of global vascular risk rather than a GDF-15-specific cerebrovascular mechanism [5,59].

For unstable angina and troponin-negative suspected ACS, the biomarker may be especially attractive, but the evidence is also most vulnerable to confounding. In such patients, an elevated GDF-15 does not prove coronary plaque instability; it more often indicates that the patient carries a substantial burden of systemic risk [52,53]. This finding may become clinically relevant in future validated post-triage pathways, but current evidence does not show that GDF-15-guided follow-up improves outcomes. In STEMI, by contrast, diagnosis is usually ECG- and reperfusion-driven; GDF-15 has no established incremental diagnostic role, but may help identify patients at increased risk of death, bleeding, or HF despite successful reperfusion [45,54].

This endpoint-specific pattern should shape how results are presented. A review or manuscript that states only that GDF-15 predicts major adverse cardiovascular events (MACE) is clinically imprecise. A composite dominated by death and HF has a different meaning from a composite dominated by recurrent MI or urgent revascularization [5,58]. For an internist or cardiologist planning care after ACS, the most useful statement is that GDF-15 appears strongest for mortality and global vulnerability, meaningful for bleeding, biologically plausible for HF, and less specific for recurrent coronary thrombosis alone [5,59,67]. This endpoint-specific pattern is summarized in Table 3.

## 7. Incremental Value Beyond Established Tools

Independent hazard ratios are not enough. A biomarker that remains statistically significant after adjustment may still fail to improve discrimination, calibration, net reclassification, or patient management [10,68]. Several NSTE-ACS analyses have shown that GDF-15 can add prognostic information beyond clinical models, hs-cTnT, natriuretic peptides, inflammatory markers, and renal function [51,68,69]. Widera and colleagues reported improved risk assessment when GDF-15 was added to the GRACE framework, and subsequent biomarker work suggested incremental value beyond GRACE and hs-cTnT in NSTE-ACS [10,68].

Compared with other established or emerging biomarkers evaluated in ACS, GDF-15 occupies a distinct but partly overlapping biological domain. High-sensitivity cardiac troponin primarily reflects myocardial injury, whereas BNP and NT-proBNP predominantly reflect hemodynamic stress and heart failure risk, and high-sensitivity C-reactive protein or interleukin-6 capture inflammatory activity. Copeptin reflects acute endogenous stress and has mainly been studied as an early diagnostic adjunct, while soluble ST2 and galectin-3 provide information related to myocardial stress, remodeling, fibrosis, and subsequent heart failure [67,70,71,72]. By contrast, GDF-15 integrates inflammatory, metabolic, renal, aging-related, and hemodynamic stress signals and appears particularly informative for mortality, frailty, and bleeding. This breadth may improve global risk stratification, but it also reduces disease specificity. GDF-15 should therefore be evaluated as an orthogonal component of multimarker models combining hs-cTn, natriuretic peptides, renal function, and GRACE 2.0 rather than as a replacement for any of these tools [60,68,69].

**Figure 2:**
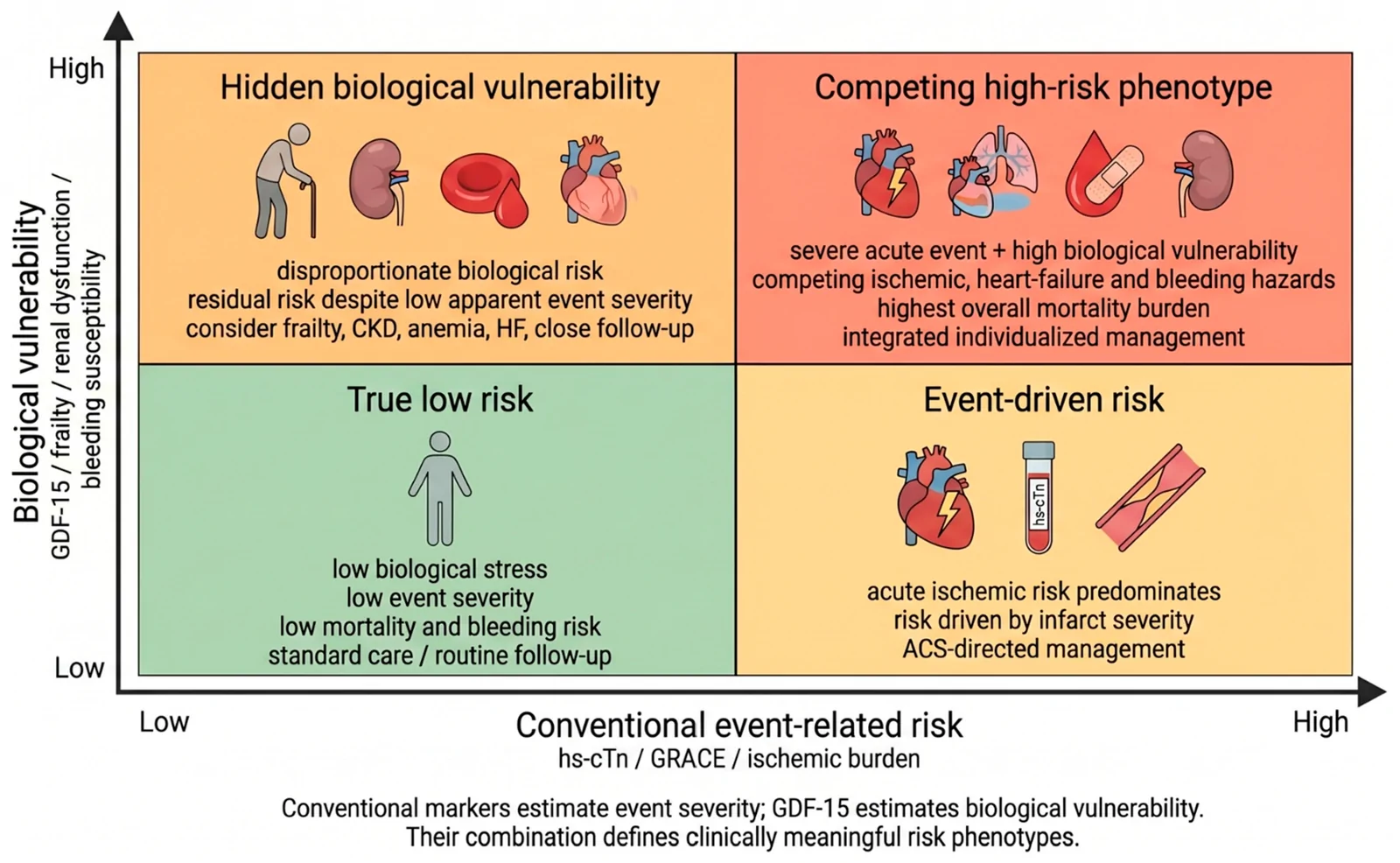
Conceptual risk phenotype matrix separating conventional event-related risk from biological vulnerability. The matrix is illustrative and has not been prospectively validated. The matrix represents a conceptual interpretation of the endpoint-specific evidence summarized in Table 3 and does not constitute a validated risk-classification model.

The GRACE 2.0 era raises the evidentiary bar. Lenell and colleagues studied 751 ACS patients with baseline GDF-15 and GRACE 2.0 data and followed them for all-cause mortality. GDF-15 alone had stronger three-year mortality discrimination than GRACE 2.0 (time-dependent AUC 0.82 [95% CI 0.75–0.89] versus 0.76 [95% CI 0.67–0.84]), and adding GDF-15 to GRACE 2.0 improved the long-term C-index from 0.74 to 0.76 at a median of 6.4 years [60]. Those data are clinically interesting, but the gain is moderate and not practice-changing by itself. They support further integration of GDF-15 with GRACE 2.0; they do not establish GDF-15-guided discharge, invasive timing, surveillance intensity, or antithrombotic selection [11,60]. Risk reclassification must be linked to a management decision: earlier follow-up, HF assessment, renal review, geriatric evaluation, bleeding surveillance, or a different antithrombotic strategy [10,11]. Statistical association is not equivalent to clinical utility. One way to conceptualize the potential incremental role of GDF-15 is to separate event-driven risk from broader biological vulnerability, as illustrated in Figure 2.

The most useful incremental value studies will therefore be those that report calibration, clinically meaningful risk strata, and decision curves, not only the AUC [11,73]. A biomarker can improve average discrimination while miscalibrating risk in older adults, patients with CKD, or frail women, and those are exactly the patients in whom GDF-15 is likely to be high [5,10]. Net reclassification is also fragile unless the categories correspond to decisions [10,51]. Moving a patient from an estimated 4% to 6% long-term mortality risk may be statistically correct, but it matters only if it changes follow-up intensity, surveillance, or therapy in a way that benefits the patient [1,11]. Although the ABC-ACS model underwent external validation in the TRACER cohort and was evaluated using decision curve analysis [11], no prospective impact study has demonstrated that adding GDF-15 to a contemporary ACS care pathway changes management and improves patient outcomes [11]. The available evidence therefore supports statistical incremental value in selected cohorts, but not outcome-validated clinical re-stratification.

## 8. GDF-15, Bleeding Risk and Antithrombotic Decision-Making

Bleeding is the most distinctive therapeutic risk domain for GDF-15. In the PLATO biomarker substudy, baseline GDF-15 was measured in 16,876 patients and was independently associated with major non-CABG bleeding as well as ischemic and mortality outcomes. For each one standard deviation increase in log-transformed GDF-15, the adjusted hazard ratio was 1.37 for major bleeding, 1.41 for cardiovascular death, 1.41 for all-cause death, 1.15 for spontaneous MI, and 1.19 for stroke [59]. A separate PLATO analysis found that GDF-15 measured one month after ACS was associated with subsequent major bleeding during DAPT [74]. These findings support GDF-15 as a prognostic bleeding risk enrichment biomarker, but not as a treatment selection biomarker [6,59,74]. No prospective interventional or randomized study has shown that modifying DAPT duration, P2Y12 inhibitor intensity, de-escalation, or intensified antithrombotic therapy according to GDF-15 improves net clinical outcomes; risk prediction must therefore be distinguished from treatment selection evidence [1,67,75,76,77,78].

Established bleeding risk assessment should continue to rely on clinical frameworks such as ARC-HBR and PRECISE-DAPT, together with clinical judgment [79,80,81]. Within the literature reviewed, we did not identify a head-to-head study demonstrating that GDF-15 improves clinically meaningful bleeding classification beyond ARC-HBR or PRECISE-DAPT sufficiently to alter antithrombotic management. Although GDF-15 may summarize several relevant domains, including age, renal dysfunction, anemia, prior bleeding, cancer, anticoagulation, and frailty, it should not independently shorten or intensify antiplatelet therapy [6,79,80,81]. Its current role is limited to research enrichment and hypothesis generation, including multimarker analyses and future randomized biomarker-guided trials [11,82].

## 9. Serial GDF-15 After ACS: From Admission Biomarker to Trajectory Biomarker

A single admission biomarker is attractive operationally, but ACS risk is dynamic. BIOMArCS is therefore important. In a nested case control analysis, serial GDF-15 values after ACS were higher in patients who later developed recurrent ACS or cardiovascular death than in matched controls; values were relatively stable from 30 days onward, a pattern consistent with persistent post-discharge biological risk rather than acute index event noise [44]. In the larger serial BIOMArCS analysis, 844 patients contributed more than 12,000 blood samples; after adjustment including a six-month post-discharge GRACE score, serial log GDF-15 was associated with cardiovascular death or recurrent ACS, with a hazard ratio of 1.81 for each one standard deviation increase [61]. Trajectory interpretation remains uncertain. A rising GDF-15 may reflect vascular instability, but it may equally reflect worsening HF, renal decline, systemic inflammation, cachexia, or intercurrent disease [46,61,83]. Serial monitoring may eventually be useful for post-discharge surveillance, especially in patients whose clinical risk appears intermediate, but no trial has shown that changing care according to a GDF-15 trajectory improves outcomes [44,61]. For now, serial GDF-15 is a research tool and a useful conceptual model for residual risk after troponin-based triage [44,83]. The BIOMArCS cohort profile and subsequent repeated biomarker analyses support the broader methodological concept of trajectory-based post-ACS risk assessment [83,84]. A clinically credible serial strategy would need predefined responses. For example, a persistently high or rising post-ACS GDF-15 might trigger assessment for occult HF, renal deterioration, anemia, inflammatory disease, medication intolerance, or frailty, rather than an automatic coronary angiogram [46,61]. That distinction matters. Serial GDF-15 may be a surveillance marker for deteriorating biology; it is not yet an event localizing marker [5,44]. The intervention attached to the signal must therefore be broad enough to match the biology but specific enough to avoid unnecessary testing [61,83].

Any response to persistent or rising post-discharge GDF-15 should therefore be framed as a non-validated, hypothesis-generating assessment framework rather than a standardized clinical algorithm. A research workflow would first confirm the assay platform, sampling interval, and analytical comparability; second, exclude acute hemodynamic instability or intercurrent systemic illness; third, reassess HF status, eGFR, hemoglobin, inflammatory disease, frailty, nutrition, and weight loss; and fourth, avoid automatic coronary angiography or DAPT modification based on the biomarker alone. Subsequent management should be directed by the clinical disorder identified during reassessment, not by the GDF-15 trajectory itself.

## 10. Residual Risk After hs-cTn-Based Exclusion of AMI

Meeting validated hs-cTn rule-out criteria supports safe exclusion of AMI, but it does not imply low long-term biological or cardiovascular risk [1,7]. A patient who meets rule-out criteria may still be elderly, frail, renally impaired, inflamed, anemic, or close to HF decompensation [7,52]. Chest-pain cohorts without AMI show that higher GDF-15 is associated with increased long-term risk of death, cardiovascular death, and HF hospitalization despite exclusion of index infarction [52]. STOP-CP data similarly suggest that GDF-15 was not independently associated with the index visit composite after adjustment, but became associated with 30- and 90-day death or AMI, a pattern consistent with delayed residual risk detection rather than acute diagnostic rule-in [53].

The clinical consequence should be proportionate. GDF-15 should not determine ED discharge alone [53,64]. This strategy could be evaluated prospectively as a means of selecting patients for closer follow-up, but GDF-15 should not currently determine emergency department disposition. This is the practical space where the biomarker may matter: not deciding whether the troponin is positive, but questioning whether the patient is truly at low risk [7,63].

After AMI has been excluded, persistent or recurrent symptoms should be evaluated according to contemporary chronic coronary syndrome guidelines, independently of GDF-15 measurement [85].

**Figure 3:**
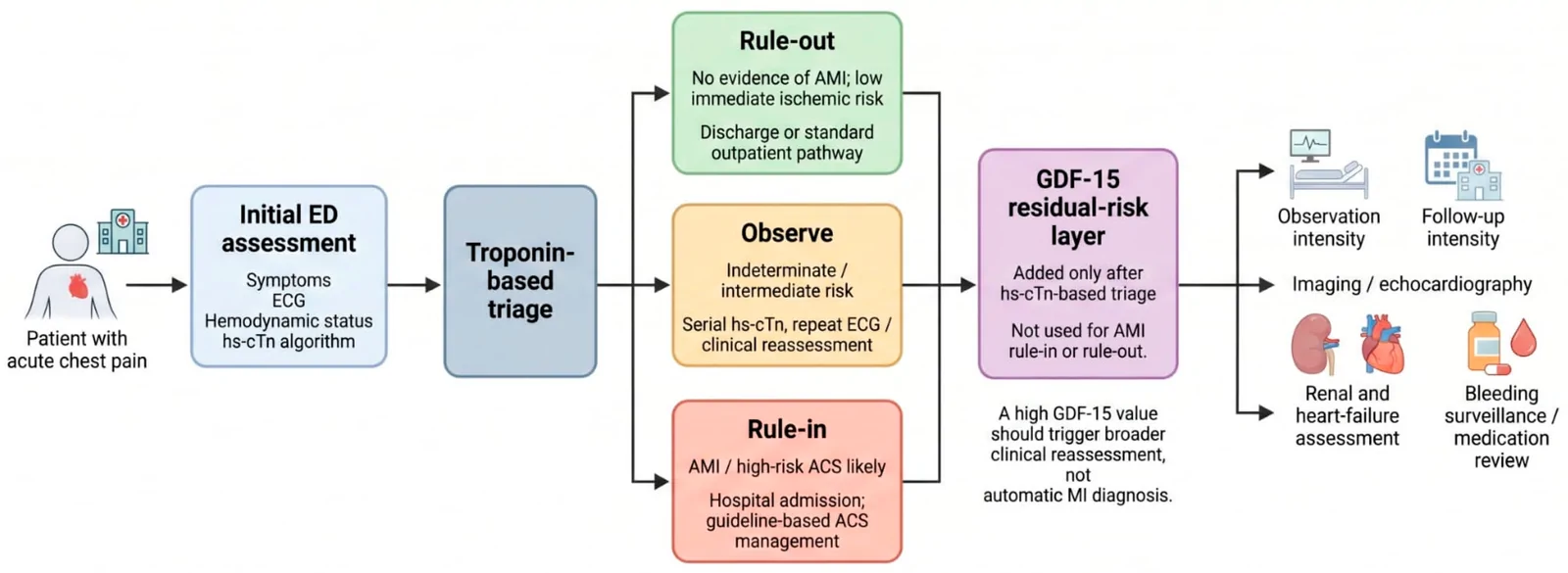
Conceptual, non-validated clinical pathway positioning GDF-15 after hs-cTn-based triage. This hypothesis-generating framework should not independently determine diagnosis, disposition, or treatment. The pathway synthesizes the implementation constraints discussed in Section 7, Section 8, Section 9, Section 10, Section 11, Section 12 and Section 13 and does not represent an outcome-validated clinical algorithm.

After AMI has been excluded, an elevated GDF-15 concentration should be interpreted as a non-specific vulnerability signal rather than as evidence of ACS [5,52]. If GDF-15 is measured in a research or selected clinical context, the result may justify clinically driven assessment for renal dysfunction, left ventricular dysfunction, anemia, poor functional reserve, uncontrolled diabetes, or systemic inflammatory disease [7,52]. It should not by itself determine emergency department disposition or treatment [53,63,86]. A proposed ED pathway that preserves hs-cTn-based MI triage while positioning GDF-15 as a downstream residual risk layer is shown in Figure 3.

## 11. Clinical Implementation: Where Could GDF-15 Actually Change Management?

Current ACS guidelines do not support routine GDF-15 testing for prognostic purposes. The 2023 ESC ACS guideline acknowledges that biomarkers such as GDF-15 may provide prognostic information, but states that their assessment has not been shown to improve patient management and that their added value beyond GRACE-based risk calculation and natriuretic peptides appears marginal [86]. Therefore, GDF-15 should currently be viewed as a candidate implementation biomarker rather than a guideline-mandated component of ACS care [5,86].

A realistic implementation pathway for GDF-15 is likely to be model-based rather than threshold-based. The ABC-ACS ischemia risk score combines age, GDF-15, NT-proBNP, the extent of coronary artery disease, prior vascular disease, Killip class, ACS type, and the P2Y12 inhibitor used [11]. The score was externally validated in the TRACER cohort and evaluated using decision curve analysis [11]. However, broader independent validation and recalibration across contemporary healthcare systems, prospective impact assessment, and evidence that model-guided care improves outcomes are still required [14,86].

There are six plausible use cases. First, after ED hs-cTn triage, GDF-15 could act as a residual risk layer, particularly in older or multimorbid patients [53,87]. Second, in confirmed NSTEMI with intermediate GRACE or GRACE 2.0 risk, it could refine long-term mortality estimates and prompt more systematic assessment of ventricular function, renal function, and frailty [11]. Third, in elderly ACS patients with CKD, it may reveal biological vulnerability but cannot separate the risk signal from confounding unless interpreted clinically [5,11]. Fourth, in patients with competing ischemic and bleeding hazards, it may enrich benefit–risk discussion but cannot replace the Academic Research Consortium for High Bleeding Risk (ARC-HBR), the PRECISE-DAPT score, or clinical judgment [59,80,81]. Fifth, after discharge, it may identify patients for closer follow-up and HF surveillance [46,52,61]. Sixth, in DAPT duration or de-escalation research, it may help define a phenotype for randomized testing [11,81]. These potential use cases, together with the decisions they might affect and the evidence still required before implementation, are summarized in Table 4.

The boundary is critical. For GDF-15 to achieve clinical utility, future biomarker-guided studies must link measurement to a prespecified action, such as monitoring intensity, timing of review, imaging, renal or HF optimization, bleeding surveillance, or enrollment into a biomarker-guided trial [11,88]. Without such a link, measurement risks biomarker inflation without decision impact [11,89,90]. The most immediately testable implementation model is selective rather than universal use: the older patient in the observation zone after hs-cTn testing, the NSTEMI patient with intermediate GRACE 2.0 risk but substantial comorbidity, or the post-PCI patient in whom ischemic and bleeding risk both appear high [11,81]. In these settings, GDF-15 could function as an additional risk enrichment layer, provided the pathway specifies what a high value does and does not authorize [11,86,88].

## 12. Threshold Calibration, Cut-Offs and Interpretation

Historical studies of GDF-15 in ACS and post-ACS populations frequently used three research categories: <1200 pg/mL, 1200–1800 pg/mL, and >1800 pg/mL. These categories demonstrated graded prognostic associations within the populations in which they were studied, but they are not validated clinical decision limits and should not be interpreted as universally applicable low-, intermediate-, or high-risk strata. Their meaning depends on assay platform, age, renal function, HF status, sampling time, clinical setting, and treatment era [5,8,43,49,56,57,58]. In an individual patient data meta-analysis of 53,486 participants from eight clinical trials, the risk of cardiovascular death and HF hospitalization increased across these historical categories [5]. In PROVE IT-TIMI 22, the two-year rates of death or MI were 5.7%, 8.1%, and 15.1% across the <1200, 1200–1800, and >1800 pg/mL groups, respectively [8]. MERLIN-TIMI 36 similarly reported adjusted hazard ratios for cardiovascular death or HF hospitalization of 1.55 for 1200–1800 pg/mL and 1.94 for ≥1800 pg/mL relative to <1200 pg/mL [43]. During a median follow-up of 4.9 years (IQR 4.2–5.8; maximum 6.5 years), a concentration >1800 pg/mL was associated with all-cause mortality (HR 4.09; 95% CI 1.57–10.71) in a single-center ACS cohort [58]. In a chest pain population without confirmed infarction, GDF-15 ≥1200 pg/mL measured three months after hospitalization defined a group with higher observed mortality, but this cohort-specific association likewise does not establish a bedside treatment threshold [46].

Studies of patients presenting to the ED with acute chest pain have tested somewhat different cut-offs, reflecting a distinct population and clinical objective. Schaub et al. demonstrated that GDF-15 had moderate diagnostic accuracy for MI (AUC 0.69) but high discriminatory performance for all-cause mortality (AUC 0.85), outperforming hs-cTnT and BNP [50]. Tzikas et al. confirmed that in a population of 1818 patients with suspected MI, median GDF-15 in those with adverse outcomes was 1660 pg/mL compared with 756.6 pg/mL in event-free patients, and GDF-15 significantly reclassified risk beyond the GRACE score (categorical NRI 12.5%; IDI 14.56%; 95% CIs not reported) [51]. In the STOP-CP study (1428 patients from eight US EDs), GDF-15 was independently associated with the composite endpoint of death/MI at 30 days (adjusted OR 1.27 per SD) and 90 days (OR 1.55 per SD), but not during the index visit, a pattern consistent with a short-term prognostic association rather than acute diagnostic rule-in [53]. The most recent multicenter study by Crisanti et al. (4779 patients) confirmed only modest diagnostic accuracy of GDF-15 for index MI/NSTE-MI at presentation (AUC 0.69), but demonstrated high discriminatory performance for all-cause mortality at both 90 days (C-index 0.86) and five years (C-index 0.84), outperforming hs-cTnT and NT-proBNP at the five-year horizon [7]. These values—including an optimized threshold of approximately 1299 pg/mL for predicting six-month death or MI in a suspected MI cohort [51], an upper-quartile threshold of approximately 1430 pg/mL in patients without AMI [52], and an exploratory threshold of 1371 pg/mL in STOP-CP [53]—are useful for interpreting the literature, but do not constitute universal bedside decision thresholds. The main GDF-15 cut-offs used across ACS and suspected-ACS studies, and their current implementation status, are summarized in Table 5.

Within-subject biological variation in GDF-15 is relatively low (coefficient of variation of approximately 6.3%), making this biomarker potentially useful for monitoring changes over time, with a reference change value (RCV) of approximately 23% [47]. However, between-assay variability and the lack of standardization across measurement platforms (immunoassays from different manufacturers and emerging mass spectrometry techniques) remain significant barriers to the clinical implementation of universal thresholds [26]. The missense variant rs1058587 (p.H202D) in the GDF-15 gene may affect epitope binding and cause significant heterogeneity of results between assays [19,91].

Before GDF-15 can be used across institutions, cross-platform commutability studies, common reference materials, transparent reporting of assay calibration, and platform-specific clinical decision limits will be required. Until such harmonization is achieved, concentrations and proposed thresholds generated with one analytical method should not be assumed to be directly transferable to another [26,41,42].

## 13. Cardiorenal, Multisystem, and Multimarker Interpretation

Renal dysfunction is not merely a covariate to be adjusted away when interpreting GDF-15. Circulating concentrations rise as eGFR declines [92,93], but experimental and human biopsy-linked data also indicate that renal tissue stress and intrarenal GDF-15 expression may contribute directly to the circulating signal. In two CKD cohorts, higher plasma GDF-15 was associated with subsequent eGFR decline or kidney failure independently of baseline kidney function, albuminuria, and comorbidity; urinary GDF-15 has additionally been related to renal histopathology and adverse renal outcomes [94,95]. Accordingly, CKD can act simultaneously as a confounder, a biological source of GDF-15, and a component of the predicted prognosis.

For ACS patients with impaired renal function, the practical consequence is that a value above 1200 or 1800 pg/mL cannot be interpreted using the same baseline risk assumptions as in a younger patient with preserved eGFR. No mathematical correction, eGFR-stratified reference interval, or CKD-specific ACS decision threshold has been prospectively validated. GDF-15 should therefore be interpreted jointly with eGFR, albuminuria, where available, hemoglobin, natriuretic peptides, HF status, age, and frailty. Until CKD-specific calibration and prospective validation are available, an elevated concentration in this population should be treated as a composite cardiorenal vulnerability signal rather than an ACS-specific treatment threshold.

The same GDF-15 concentration can therefore represent markedly different biological states. In an older patient with CKD, chronic HF, anemia, frailty, malignancy, or systemic inflammation, a high value may predominantly reflect chronic multisystem disease and competing non-coronary mortality risk. In a younger patient with preserved renal function and no major comorbidity, the same value is more unusual but still does not localize the source of stress. Statistical adjustment does not create a validated patient-level correction formula, and no current method can partition an individual result into components attributable to the index ACS, kidney dysfunction, HF, diabetes, inflammation, cancer, or frailty. Fixed thresholds may therefore overclassify older and multimorbid patients, while a strong association with all-cause mortality may partly reflect non-cardiovascular risk rather than modifiable coronary risk [5,26,30,31,32,39,94,95]. A concentration of 1900 pg/mL would be more unusual in a younger patient with preserved renal function than in an older patient with CKD and heart failure; however, no validated age- or eGFR-specific interpretation allows a management decision to be made from either value alone.

GDF-15 should also be placed within a wider multisystem signaling network rather than viewed as an isolated cardiovascular biomarker [91]. Bone- and mineral-metabolism mediators, including osteocalcin, the osteoprotegerin/RANKL axis, osteopontin, fibroblast growth factor 23 (FGF23), sclerostin, and Klotho, connect skeletal remodeling, vascular inflammation and calcification, renal dysfunction, metabolic disease, and cardiovascular outcomes. In NSTE-ACS, osteoprotegerin has been associated with cardiovascular mortality and HF, and in PLATO it was associated with major bleeding rather than ischemic events after adjustment [96,97]. Osteopontin rises after acute MI, although small studies have not established a reliable prognostic role [98]. In cardiorenal and diabetic cohorts, GDF-15 and FGF23 have provided partly complementary mortality and renal risk information, while observational studies of Klotho, sclerostin, and osteocalcin have produced population-dependent and sometimes discordant associations [99,100,101,102]. These data provide biological context for multisystem vulnerability but do not justify adding these mediators to routine ACS pathways.

Future implementation is therefore more likely to rely on a parsimonious, externally validated multimarker model than on GDF-15 as a stand-alone test. The components should represent complementary domains: myocardial injury (hs-cTn), hemodynamic stress (BNP or NT-proBNP), renal function (eGFR and, where relevant, albuminuria), inflammation, and systemic vulnerability, while avoiding redundant measurements. Multimarker studies in ACS support the combination of complementary biological domains [67,68,69], while studies in atrial fibrillation, population cohorts, and diabetes illustrate the broader feasibility of multimarker approaches outside ACS [103,104,105]. However, any panel intended for ACS implementation must demonstrate calibration, decision curve benefit, analytical feasibility, cost-effectiveness, and a prespecified management consequence beyond established clinical scores. A larger biomarker panel is not inherently superior; the clinically relevant standard is whether a compact model improves decisions and outcomes.

## 14. Limitations of the Evidence

The evidence base is strong for association but weak for clinical actionability. No adequately powered ACS trial has assigned patients to a discharge strategy, invasive approach, follow-up pathway, bleeding surveillance strategy, or DAPT duration according to GDF-15, and most available evidence is observational or derives from biomarker substudies [5,6,56,62,106]. The detailed limitations related to confounding, reverse causation, selection, assay and sampling variability, outcome adjudication, and publication bias are considered below.

### Critical Appraisal of Evidence Quality

Most clinical evidence is observational or derives from biomarker substudies in which randomization applied to treatment, not to biomarker-guided care. Associations may therefore reflect residual confounding by age, CKD, HF, diabetes, anemia, inflammation, malignancy, frailty, socioeconomic factors, and treatment intensity. Multivariable adjustment cannot remove unmeasured disease severity or measurement error. Reverse causation is also plausible: an elevated GDF-15 concentration may be a consequence of occult HF, renal deterioration, catabolic illness, cancer, or frailty that already increases mortality, rather than a causal mediator whose reduction would improve outcome [5,6,13,26,90].

Selection bias differs by design. Trial biomarker substudies include patients meeting trial eligibility criteria and often require an available stored sample and survival to the sampling time, whereas ED cohorts, post-ACS survivor cohorts, and serial measurement studies select different risk sets. Loss to follow-up, missing specimens, complete case analysis, and survivorship to convalescent sampling can further alter observed associations. These populations and estimates should not be treated as directly interchangeable [13,14].

Outcome measurement is also heterogeneous. Studies differ in the adjudication and composition of index MI, spontaneous MI, recurrent ACS, HF hospitalization, cardiovascular death, and major bleeding endpoints. Differences in endpoint definitions can change effect estimates independently of biomarker performance. Between-platform assay differences, epitope recognition, sampling time, sample storage, and data-derived cut-offs create additional exposure misclassification and limit the transfer of thresholds between studies [17,18,19,26,41,42].

Publication and selective-reporting bias are also likely because positive biomarker associations and favorable reclassification statistics are more likely to be published than null incremental analyses, poor calibration, or non-actionable decision curves. As a narrative review without a formal meta-analysis, publication bias could not be quantified statistically. Consequently, the direction of association is more secure than the magnitude of incremental value or readiness for implementation [89,90]. Evidence from broader coronary artery disease cohorts may support biological consistency but cannot substitute for contemporary ACS-specific implementation studies [107]. Category-free NRI can yield numerically large values even when gains in discrimination or clinical decision-making are limited and should not be interpreted as equivalent to clinically anchored categorical reclassification [108].

Geographic and ethnic transportability also remains incompletely established. Evidence from Asian populations is comparatively limited and derives mainly from Japanese and Chinese cohorts encompassing broader ischemic heart disease or coronary artery disease populations rather than exclusively contemporary ACS cohorts. A Japanese cohort of 632 patients with ischemic heart disease associated higher GDF-15 with all-cause mortality, MACE, HF rehospitalization, bleeding, and accumulation of the Japanese high-bleeding risk criteria, but not with thrombotic events after adjustment [109]. In the prospective five-center Chinese BIPass registry of 6322 patients with CAD, admission GDF-15 was associated with cardiovascular death, HF events, cardiogenic shock, and clinically significant bleeding, but not MI or stroke [110]. A separate prospective Chinese CAD cohort also supported long-term prognostic associations with MACE and all-cause death [111]. These studies support consistency in the direction of prognostic associations across Asian settings but do not establish ethnicity-specific or region-specific clinical decision thresholds; external calibration across geographic, ethnic, clinical, and analytical settings remains necessary.

## 15. Research Agenda

The next phase should not be another series of small association studies [90,106]. Needed studies include prospective validation in hs-cTn-era ED cohorts, direct integration with GRACE 2.0, external calibration across healthcare systems, decision curve analyses using clinically meaningful thresholds, and health economic evaluation [7,10,73]. High-bleeding risk ACS cohorts should test whether GDF-15 improves ARC-HBR and PRECISE-DAPT phenotyping [59,80,81]. DAPT duration, de-escalation, and intensified antithrombotic strategies should be studied only in designs that separate risk prediction from treatment effect prediction [6,56].

Serial biomarker trials should test whether post-discharge GDF-15 trajectories can trigger effective interventions: earlier HF assessment, renal optimization, rehabilitation, frailty care, bleeding surveillance, or intensified secondary prevention [44,46,61]. The key future question is simple: can GDF-15-guided care improve outcomes, or does it merely reclassify risk [6,26,106]? Prospective implementation studies should also compare a parsimonious integrated panel against GDF-15 alone and established clinical models, with attention to calibration, net benefit, cost, and treatment consequences.

## 16. Conclusions

Taken together, GDF-15 should be viewed primarily as a biomarker of biological vulnerability and residual risk after ACS, rather than as a biomarker of myocardial necrosis or a stand-alone diagnostic test for AMI. Its strongest and most consistent clinical signal concerns mortality, heart failure, bleeding, and broader post-ACS vulnerability. The path to clinical use requires integration with hs-cTn, GRACE 2.0, bleeding risk models, and management pathways. Implementation will be justified only when risk reclassification is linked to a defensible clinical action and when measuring GDF-15 improves care, not merely prediction. Accordingly, current evidence establishes GDF-15 primarily as a prognostic and risk-enrichment biomarker; it does not establish that GDF-15-guided management improves clinical outcomes.

## Figures and Tables

**Table 1:** Key clinical studies of GDF-15 in ACS and suspected ACS.

Study and Cohort	Study Type and Sampling	Follow-Up and Endpoint	Main Adjusted Result	Clinical Interpretation	Reference
Emergency-department and suspected-ACS cohorts					
**Schaub et al., 2012** ED chest pain; suspected ACS (AMI 18%); n = 646	Prospective; international multicenter; ED cohort; presentation sampling; continuous analysis; no fixed cut-off.	Median 26 mo; index AMI and all-cause death.	AMI AUC 0.69 vs hs-cTnT 0.96; mortality AUC 0.85; categorical mortality NRI 15% (95% CI not reported).	Weak for AMI diagnosis; useful for prognosis. No validated rule-in/rule-out pathway.	[50]
**Tzikas et al., 2017** Suspected MI; n = 1818	Prospective; three-center; Suspected-MI cohort; baseline sampling; optimized 1298.9 pg/mL cut-off and continuous Cox models.	6 mo; death or MI.	Adjusted HR 1.57 after GRACE covariates; age/sex model C-index approximately 0.80.	Prognostic study; not designed for index-AMI diagnosis.	[51]
**Myrmel et al., 2023** Chest pain after exclusion of index AMI; n = 1320	Prospective observational; Norwegian; Post-rule-out chest-pain cohort; baseline sampling with hs-cTnT; upper-quartile and cross-classification analyses.	Median 1523 d; death, CV death, HF hospitalization.	Highest category: death HR 2.75, CV death HR 3.74, HF hospitalization and HR 2.60; mortality discrimination improved.	Residual risk marker after a negative AMI work-up; not an AMI diagnostic test.	[52]
**Crisanti/APACE, 2026** ED chest pain; final AMI vs alternative diagnosis; n = 4779	Prospective; international multicentre; ED chest-pain cohort; presentation sampling; integrated with hs-cTn pathways; continuous modeling.	90 d and 5 y; index MI/NSTE-MI and all-cause death.	Index MI/NSTE-MI AUC 0.69; mortality C-index 0.86 at 90 d and 0.84 at 5 y.	Strong prognostic discrimination; no role as an hs-cTn substitute.	[7]
**Mumma/STOP-CP, 2025** Suspected ACS across 8 US EDs; n = 1428	Prospective; US; Multicentre ED cohort; baseline sampling; per-SD and tertile analyses.	Index visit, 30 d, and 90 d; death or AMI.	No independent index-event association; per-SD OR 1.27 at 30 d and 1.55 at 90 d.	Delayed residual risk signal; insufficient for acute rule-in/rule-out decisions.	[53]
Established ACS and post-MI cohorts					
**Wollert et al., 2007** NSTE-ACS; n = 2081	International multicentre randomized-trial biomarker substudy; NSTE-ACS cohort; historical research categories: <1200, 1200–1800, >1800 pg/mL.	1 y; mortality.	Mortality increased stepwise; >1800 pg/mL was associated with higher risk beyond clinical and biomarker predictors.	Pre-hs-cTn era; thresholds require contemporary validation.	[49]
**Kempf et al., 2007** Trial-based STEMI; n = 741	International multicentre randomized-trial biomarker analysis; trial-based STEMI cohort; baseline sampling; categorical and continuous analyses.	30 d to 1 y; death and composite events.	Provided incremental prognostic information beyond clinical and biochemical markers.	Older reperfusion era; limited transferability to contemporary primary PCI.	[54]
**Khan et al., 2009** Survivors of acute MI; n = 1142	Prospective; single-center UK; post-MI cohort; early post-MI sampling; continuous and categorical analyses.	Median approximately 1.3 y; death and HF.	Was associated with death and HF after multivariable clinical and biomarker adjustment.	No actionable threshold; treatment era limits generalizability.	[55]
**Wollert/FRISC II, 2007** Randomized NSTE-ACS strategy substudy; n = 2079	Prospective; Scandinavian multicentre randomized trial substudy; randomized NSTE-ACS strategy substudy; historical research categories: <1200, 1200–1800, >1800 pg/mL.	2 y; death/MI and invasive-strategy interaction.	Higher GDF-15 was associated with greater risk and an exploratory interaction with invasive strategy.	Hypothesis-generating; treatment was not biomarker-randomized.	[56]
**Damman/ICTUS, 2014** NSTE-ACS invasive-strategy substudy; n = 1151	Prospective; Dutch multicentre randomized-trial substudy; NSTE-ACS invasive-strategy substudy; historical research categories: <1200, 1200–1800, >1800 pg/mL.	Long-term; death or spontaneous MI.	Association was mainly mortality-driven; IDI improved, but NRI was limited.	Mixed incremental statistics in an older trial context.	[57]
**Bonaca/PROVE IT, 2011** Stabilized post-ACS trial cohort; n = 3501	Prospective; international multicentre randomized trial biomarker substudy; post-ACS trial cohort; post-stabilization baseline categories.	2 y; death, MI, and HF.	>1800 pg/mL was associated with higher death/MI risk and enriched HF risk beyond TIMI variables and biomarkers.	Applies after stabilization; not to ED triage.	[8]
**Peiro et al., 2019** Consecutive ACS cohort; n = 358	Single-center Spanish observational cohort; consecutive ACS cohort; baseline sampling; >1800 pg/mL.	Long-term; all-cause death.	>1800 pg/mL was associated with all-cause death (adjusted HR 4.09) after GRACE, LVEF, and comorbidity adjustment.	External validation and treatment-linked thresholds are lacking.	[58]
**Hagstrom/PLATO, 2016** ACS biomarker substudy; ticagrelor vs clopidogrel; n = 16,876	Prospective; international multicentre randomized trial biomarker substudy; randomized trial biomarker substudy; central-laboratory baseline; per-SD log increase and categories.	Trial follow-up; death, MI, stroke, major bleeding.	Per-SD log increase: major bleeding HR 1.37; CV and all-cause death HR 1.41; weaker MI/stroke signals.	No established treatment-effect interaction with antiplatelet strategy.	[59]
**Lenell et al., 2026** Prospective ACS cohort with echocardiography; n = 751	Prospective Swedish TOTAL-AMI cohort; prospective ACS cohort with echocardiography; baseline sampling; added continuously to GRACE 2.0.	3 y and median 6.4 y; all-cause death.	3-y AUC 0.82 vs 0.76 for GRACE 2.0; long-term C-index 0.76 vs 0.74 after adding GDF-15.	Incremental gain was modest; no management-linked validation.	[60]
Serial post-discharge biomarker studies					
**Buljubasic/BIOMArCS, 2019** Nested case-control study; 37 cases/74 controls	Prospective Dutch multicentre parent cohort; nested case control study; repeated post-discharge sampling; trajectory analysis.	1 y; CV death or recurrent ACS.	Cases had persistently higher GDF-15 from day 30 onward after matching and clinical adjustment.	Small event count; mechanism of serial elevation remains uncertain.	[44]
**Gurgoze/BIOMArCS, 2023** Post-ACS serial cohort; n = 844; 12,218 samples	Prospective observational; Dutch multicentre; Serial post-ACS cohort; median 17 samples/patient; joint serial modeling.	1 y; CV death or recurrent ACS.	Serial log GDF-15 was associated with the composite endpoint (adjusted HR 1.81 per SD) after GRACE adjustment.	Observational; no GDF-15-guided intervention.	[61]

**Note.** ACS, acute coronary syndrome; AMI, acute myocardial infarction; AUC, area under the curve; CV, cardiovascular; ED, emergency department; GDF-15, growth differentiation factor 15; GRACE, Global Registry of Acute Coronary Events; HF, heart failure; hs-cTn(T), high-sensitivity cardiac troponin (T); IDI, integrated discrimination improvement; LVEF, left ventricular ejection fraction; MI, myocardial infarction; NRI, net reclassification improvement; NSTE-ACS, non-ST-segment elevation acute coronary syndrome; OR, odds ratio; PCI, percutaneous coronary intervention; SD, standard deviation; STEMI, ST-segment elevation myocardial infarction. Study-design descriptors are provided to support qualitative evidence grading and do not constitute a formal risk-of-bias assessment.

**Table 2:** Diagnostic performance of GDF-15 in suspected ACS/AMI.

Study and Setting	Index-AMI Diagnostic Evidence	Prognostic or Incremental Evidence	Clinical Conclusion	Reference
Prospective international multicenter cohort **Schaub et al., 2012** ED chest pain; hs-cTnT and BNP	Index AMI AUC 0.69 vs hs-cTnT 0.96. No prespecified GDF-15 threshold; sensitivity, specificity, PPV, and NPV not reported.	Mortality AUC 0.85; categorical NRI 15% (95% CI not reported). No clinically useful diagnostic gain over hs-cTnT.	Weak diagnostic competitor; strong long-term risk marker.	[50]
Prospective three-center cohort **Tzikas et al., 2017** Suspected MI; GRACE and clinical risk	Index-AMI discrimination and threshold operating characteristics were not evaluated.	6-month death/MI C-index approximately 0.80; independent information after GRACE covariates.	Prognostic, not diagnostic.	[51]
Prospective Norwegian observational cohort **Myrmel et al., 2023** Index AMI excluded; hs-cTnT and risk factors	Not applicable because the cohort excluded index AMI.	Mortality C-statistic 0.86, increasing to 0.89 after the addition of GDF-15; improved risk classification.	Residual risk marker after a negative AMI work-up.	[52]
Prospective international multicenter cohort **Crisanti/APACE, 2026** ED chest pain; ECG plus hs-cTn algorithms	Index MI/NSTE-MI AUC 0.69. No prespecified rule-in/rule-out threshold or reported operating characteristics.	Mortality C-index 0.86 at 90 d and 0.84 at 5 y; no role as a troponin-pathway substitute.	Best evidence for diagnostic limitation and prognostic value.	[7]
Prospective US multicenter cohort **Mumma/STOP-CP, 2025** Eight US EDs; hs-cTnT, NT-proBNP, clinical variables	No independent index-event association. Exploratory 1371 pg/mL cut-off addressed 30-day death/AMI, not index AMI.	Independent association with 30- and 90-day death/AMI; high NPV reported only for the short-term composite.	May refine post-triage risk; cannot determine discharge alone.	[53]

**Note.** Threshold metrics refer to prespecified GDF-15 cut-offs for diagnosis of index AMI. BNP, B-type natriuretic peptide; ECG, electrocardiogram; NPV, negative predictive value; NT-proBNP, N-terminal pro-B-type natriuretic peptide; PPV, positive predictive value.

**Table 3:** Prognostic performance by endpoint and measurement scale.

Endpoint	Study and Source Population	Biomarker Scale or Comparison	Estimate (95% CI) or Model Metric	Clinical Interpretation	Reference
Continuous association measures					
All-cause death	International multicenter randomized-trial biomarker substudy; PLATO ACS biomarker substudy; n = 16,876	Per 1-SD increase in ln GDF-15	HR 1.41 (1.31–1.53)	Strong mortality association on a continuous scale.	[59]
Cardiovascular death	International multicenter randomized-trial biomarker substudy; PLATO ACS biomarker substudy; n = 16,876	Per 1-SD increase in ln GDF-15	HR 1.41 (1.30–1.53)	Magnitude closely parallels all-cause mortality.	[59]
Major non-CABG bleeding	International multicenter randomized-trial biomarker substudy; PLATO ACS biomarker substudy; n = 16,876	Per 1-SD increase in ln GDF-15	HR 1.37 (1.25–1.51)	Similar per-SD magnitude to mortality; does not predict treatment benefit.	[59]
Spontaneous MI	International multicenter randomized-trial biomarker substudy; PLATO ACS biomarker substudy; n = 16,876	Per 1-SD increase in ln GDF-15	HR 1.15 (1.05–1.26)	Weaker ischemia-specific association.	[59]
Stroke	International multicenter randomized-trial biomarker substudy; PLATO ACS biomarker substudy; n = 16,876	Per 1-SD increase in ln GDF-15	HR 1.19 (1.01–1.42)	Modest association with global vascular risk.	[59]
CV death or recurrent ACS	Prospective Dutch multicenter serial cohort; BIOMArCS serial post-ACS cohort; n = 844	Per 1-SD increase in serial log GDF-15	HR 1.81 (1.28–2.70)	Serial composite association; not directly comparable with baseline PLATO estimates.	[61]
Categorical association measures					
All-cause death	Single-center Spanish observational cohort; Peiró consecutive ACS cohort; n = 358	>1800 pg/mL versus the study reference category	HR 4.09 (1.57–10.71)	Categorical contrast; not comparable with per-SD hazard ratios.	[58]
Death, CV death, and HF hospitalization	Prospective Norwegian observational cohort; Myrmel chest-pain cohort after exclusion of index AMI; n = 1320	Highest GDF-15 quartile versus lower quartiles	All-cause death HR 2.75 (1.69–4.45); CV death HR 3.74 (1.31–10.63); HF hospitalization HR 2.60 (1.11–6.06)	Shows an association with residual systemic risk after a negative AMI work-up.	[52]
Discrimination and incremental model performance					
3-year all-cause mortality	Prospective Swedish TOTAL-AMI cohort; Lenell prospective ACS cohort; n = 751	GDF-15 alone versus GRACE 2.0	AUC 0.82 (0.75–0.89) versus 0.76 (0.67–0.84)	Discrimination statistic, not an endpoint effect estimate.	[60]
Long-term all-cause mortality	Prospective Swedish TOTAL-AMI cohort; Lenell prospective ACS cohort; n = 751	GRACE 2.0 plus GDF-15 versus GRACE 2.0	C-index 0.76 versus 0.74	Modest incremental discrimination; management impact untested.	[60]
6-month death or MI	Prospective three-center cohort; Tzikas suspected-MI cohort; n = 1818	GDF-15 added to GRACE covariates	Categorical NRI 12.5%; IDI 14.56% (95% CIs not reported)	Reclassification metrics; not comparable with hazard ratios or AUC.	[51]

**Note.** Estimates are grouped by measurement scale. Hazard ratios based on different biomarker transformations, categorical thresholds, reference groups, sampling strategies, or composite endpoints are not directly comparable. AUC, C-index, NRI, and IDI describe model performance rather than the magnitude of an endpoint association. AMI, acute myocardial infarction; AUC, area under the curve; CABG, coronary artery bypass grafting; CV, cardiovascular; HF, heart failure; IDI, integrated discrimination improvement; MI, myocardial infarction; NRI, net reclassification improvement; SD, standard deviation.

**Table 4:** Proposed research framework for clinical implementation.

Clinical Scenario	Hypothesis-Generating Role and Potential Action	Evidence Status	Key Evidence Gap	Key Reference
**A. ED suspected ACS after hs-cTn triage**	Residual risk layer after rule-out, rule-in, or observation classification; could be evaluated for effects on observation, follow-up timing, and referral intensity.	Moderate prognostic evidence; weak diagnostic evidence.	Prospective ED algorithms with calibration, decision curves, and missed-event/safety endpoints.	[7,50,51,52,53,64]
**B. Confirmed NSTEMI with intermediate GRACE/GRACE 2.0 risk**	Refine long-term mortality and vulnerability assessment; could be evaluated for selecting closer follow-up, echocardiography/HF review, and renal or frailty assessment.	Moderate evidence from NSTE-ACS and GRACE 2.0 cohorts.	External validation and prespecified management thresholds linked to outcomes.	[10,49,60,68]
**C. Older or frail patient with ACS and CKD**	Identify vulnerability not captured by troponin alone; could be evaluated as a trigger for integrated geriatric, renal, medication, and bleeding risk review.	Biologically plausible but heavily confounded by age, eGFR, HF, and frailty.	Calibration across CKD/frailty strata and analyses separating independent value from confounding.	[5,26,30,32,39]
**D. Competing ischemic and bleeding risk**	Characterize benefit–risk tension; could be studied as an adjunct to DAPT discussion and laboratory surveillance, but not as a stand-alone rule.	Strong association with bleeding and death in PLATO; no treatment-guidance trial.	Randomized or decision-analytic de-escalation studies demonstrating net clinical benefit.	[59,74,79,80,81]
**E. High residual risk after discharge**	Serial trajectory marker; could be evaluated for triggering earlier HF/renal review, rehabilitation, and intensification of secondary prevention.	Supportive but observational BIOMArCS evidence.	Interventional serial-monitoring trials with prespecified response pathways.	[44,46,61,83]
**F. Shortened, standard, or intensified DAPT**	Potential enrichment or stratification marker for antithrombotic trials; not for routine treatment selection.	Hypothesis-generating only.	Validated treatment-effect interaction and evidence of improved net clinical benefit.	[11,59,67,80,81,88]

**Note.** Evidence status is descriptive and does not represent a formal GRADE assessment. CKD, chronic kidney disease; eGFR, estimated glomerular filtration rate; NSTEMI, non-ST-segment elevation myocardial infarction. All proposed roles and actions are hypothesis-generating and should not be interpreted as current clinical recommendations.

**Table 5:** GDF-15 cut-offs used in ACS literature.

Threshold or Model	Context and Associated Outcome	Main Limitation	Interpretation	Key Reference
**<1200 pg/mL (historical lower-concentration research category)**	Historical NSTE-ACS and post-ACS trial frameworks; lower mortality and composite-event risk.	Not adjusted for age, renal function, assay platform, or contemporary treatment.	Historical research reference category; not a clinical low-risk designation.	[8,49,56,57]
**1200–1800 pg/mL (historical intermediate research category)**	NSTE-ACS, PROVE IT, and invasive strategy substudies; intermediate mortality and global risk.	Broad gray zone with no validated management action.	Historical research category; not a treatment threshold.	[8,49,56,57]
**>1800 pg/mL (historical higher-concentration research category)**	NSTE-ACS, PROVE IT, Peiro cohort, and trial analyses; higher mortality, HF, and composite event risk.	May primarily reflect age, CKD, HF, or frailty rather than ACS-specific biology.	Historical research category and literature anchor; not guideline-endorsed.	[8,49,56,57,58]
**1298.9 pg/mL**	Suspected-MI ED cohort; 6-month death or MI.	Data-derived cut-off with uncertain external validity.	Research threshold.	[51]
**1371 pg/mL**	STOP-CP suspected ACS cohort; 30-day death/AMI composite, not index AMI diagnosis.	Exploratory and not externally validated; high NPV applied only to the short-term composite.	Research threshold only.	[53]
**Approximately 1430 pg/mL**	Upper quartile in chest pain without AMI; death, CV death, and HF hospitalization.	Cohort-dependent and not an AMI diagnostic cut-off.	Hypothesis-generating for follow-up intensity.	[52]
**Tertiles or quartiles**	PLATO, STOP-CP, and chest-pain cohorts; cohort-specific prognostic categories.	Difficult to translate into standardized bedside reporting.	Cohort-specific interpretation.	[52,53,59]
**Continuous log-transformed modeling**	Modern biomarker, serial measurement, and GRACE 2.0 analyses; mortality, bleeding, and composite outcomes.	Statistically efficient but less intuitive and not directly actionable.	Preferred for model development, not stand-alone care.	[7,51,59,60,61]

**Note.** The <1200, 1200–1800, and >1800 pg/mL categories are historical research classifications rather than guideline-endorsed clinical decision thresholds. Their interpretation depends on assay platform, age, renal function, HF status, sampling time, clinical setting, and treatment era. None of the proposed cut-offs should be used as a stand-alone rule-in, rule-out, discharge, antithrombotic, or treatment threshold.

## Data Availability

No new data were created in this study. This article is a narrative review based on previously published literature identified through searches of PubMed/MEDLINE, Web of Science, and Scopus and manual screening of reference lists. All sources supporting the conclusions of this review are cited in the reference list.

## Abbreviations

The following abbreviations are used in this manuscript: .

ABC-ACS	Age, Biomarkers, and Clinical History-Acute Coronary Syndrome risk score
ACC	American College of Cardiology
ACS	Acute coronary syndrome
AHA	American Heart Association
AKT	Protein kinase B
ALK	Activin receptor-like kinase
AMI	Acute myocardial infarction
ARC-HBR	Academic Research Consortium for High Bleeding Risk
ARIC	Atherosclerosis Risk in Communities
ATF4	Activating transcription factor 4
AUC	Area under the curve
BIOMArCS	Biomarker Study to Identify the Acute Risk of a Coronary Syndrome
BNP	B-type natriuretic peptide
CABG	Coronary artery bypass grafting
CAD	Coronary artery disease
CHOP/DDIT3	C/EBP homologous protein/DNA damage-inducible transcript 3
CI	Confidence interval
CKD	Chronic kidney disease
cTnI	Cardiac troponin I
cTnT	Cardiac troponin T
CV	Cardiovascular
DAPT	Dual antiplatelet therapy
DNA	Deoxyribonucleic acid
ECG	Electrocardiogram
ECM	Extracellular matrix
ED	Emergency department
eGFR	Estimated glomerular filtration rate
EGR-1	Early growth response protein 1
eIF2α	Eukaryotic translation initiation factor 2 alpha
ERK	Extracellular signal-regulated kinase
ESC	European Society of Cardiology
FGF23	Fibroblast growth factor 23
FRASNET	Frailty and Sarcopenia Network
GDF-15	Growth differentiation factor-15
GFRAL	Glial cell-derived neurotrophic factor family receptor alpha-like
GRACE	Global Registry of Acute Coronary Events
GRADE	Grading of Recommendations Assessment, Development and Evaluation
HF	Heart failure
HHF	Heart failure hospitalization
HIF-1α	Hypoxia-inducible factor 1-alpha
HR	Hazard ratio
hs-cTn	High-sensitivity cardiac troponin
hs-cTnT	High-sensitivity cardiac troponin T
IDI	Integrated discrimination improvement
IQR	Interquartile range
ISR	Integrated stress response
J-HBR	Japanese version of high bleeding risk criteria
LVEF	Left ventricular ejection fraction
MACE	Major adverse cardiovascular events
MI	Myocardial infarction
NF-κB	Nuclear factor kappa B
NPV	Negative predictive value
NRI	Net reclassification improvement
NSAIDs	Nonsteroidal anti-inflammatory drugs
NSTE-ACS	Non-ST-segment elevation acute coronary syndrome
NSTE-MI	Non-ST-segment elevation myocardial infarction
NSTEMI	Non-ST-segment elevation myocardial infarction
NT-proBNP	N-terminal pro-B-type natriuretic peptide
OR	Odds ratio
P2Y12	Purinergic receptor P2Y12
PCI	Percutaneous coronary intervention
PET	Positron emission tomography
PI3K	Phosphoinositide 3-kinase
PLATO	Platelet Inhibition and Patient Outcomes
PPV	Positive predictive value
PRECISE-DAPT	Predicting Bleeding Complications in Patients Undergoing Stent Implantation and Subsequent Dual Antiplatelet Therapy
PRISMA	Preferred Reporting Items for Systematic Reviews and Meta-Analyses
RANKL	Receptor activator of nuclear factor kappa-B ligand
RCV	Reference change value
RET	Rearranged during transfection receptor tyrosine kinase
SCORE2	Systematic Coronary Risk Evaluation 2
SD	Standard deviation
SMAD	Suppressor of mothers against decapentaplegic
SPECT	Single-photon emission computed tomography
ST2	Suppression of tumorigenicity 2
STEMI	ST-segment elevation myocardial infarction
TGF-β	Transforming growth factor beta
TIMI	Thrombolysis in Myocardial Infarction
TβRII	Transforming growth factor beta receptor type II
